# Investigating the molecular mechanisms of the “Tianma-Gouteng” herb pair in treating Parkinson’s disease: a bioinformatics approach and density functional theory with molecular dynamics simulations validation

**DOI:** 10.3389/fbinf.2026.1796216

**Published:** 2026-04-01

**Authors:** Liping Zhou, Chenyang Fei, Quanxia Liu

**Affiliations:** 1 Department of Traditional Chinese Medicine, General Hospital of Ningxia Medical University, Yinchuan, China; 2 Ruihe Digital Technology Co., Ltd., Shenzhen, China; 3 Department of Oncology II, General Hospital of Ningxia Medical University, Yinchuan, China

**Keywords:** bioinformatics, density functional theory, molecular dynamics simulations, Parkinson’s disease, Tianma-Gouteng

## Abstract

Parkinson’s disease (PD) is a complex neurodegenerative disorder for which current treatments are often symptomatic and lack disease-modifying effects. The traditional Chinese medicine herb pair Tianma-Gouteng, composed of *Gastrodia elata* Bl (Tianma) and *Uncaria rhynchophylla*(Miq.) Miq. ex Havil. (Gouteng), has demonstrated clinical efficacy in treating PD motor symptoms, yet its multi-target mechanisms remain unclear. This study employs an integrated approach combining bioinformatics and computational chemistry to elucidate these mechanisms and identify key active components. Methods involved network pharmacology to identify active compounds and PD-related targets, followed by protein-protein interaction network analysis and functional enrichment. Molecular docking and 100-ns molecular dynamics (MD) simulations were utilized to evaluate the binding stability and dynamics of core component-target complexes. Additionally, Density Functional Theory (DFT) was conducted to analyze the electronic properties and reactivity of key compounds. Network pharmacology analysis identified 42 active components and 261 PD-related targets. Core targets identified were AKT1, TP53, and STAT3, which are involved in the regulation of PI3K-AKT signaling, mitochondrial apoptosis, and neuroinflammation. MD simulations demonstrated that quercetin (QU) and kaempferol (KA) formed highly stable complexes with AKT1 and TP53, exhibiting low average root-mean-square deviation (RMSD <0.2 nm), stable radius of gyration (Rg fluctuation <0.05 nm), and sustained protein-ligand hydrogen bonds. In contrast, complexes with 4–4′-hydroxybenzyloxy and 20-hexadecanoylingenol showed conformational instability, consistent with higher entropy penalties. DFT calculations revealed that QU and KA possess low HOMO-LUMO gaps, indicating high chemical reactivity, along with strong nucleophilic regions and intramolecular hydrogen bonds that facilitate target binding. The Tianma-Gouteng pair exerts anti-PD effects through the synergistic modulation of AKT1-mediated PI3K-AKT signaling, STAT3-driven neuroinflammation, and TP53-regulated apoptosis. Quercetin and kaempferol are identified as pivotal components due to their stable target binding and favorable electronic properties, providing a promising foundation for the development of novel PD therapeutics.

## 1 Introduction

Parkinson’s disease (PD) is a common neurodegenerative disorder primarily characterized by motor dysfunctions, including resting tremor, muscle rigidity, bradykinesia, and postural instability (Armstrong and Okun, 2020). The etiology of PD is multifaceted, involving a complex interplay of genetic predispositions, environmental factors, and neuroinflammatory processes. Aside from surgical interventions, there are currently no effective pharmacological treatments for PD, necessitating the urgent need for further investigation into its pathogenesis and the development of novel therapeutic strategies. Recently, natural plant extracts have garnered significant attention, and treatment approaches for Alzheimer’s disease (AD) based on modern pharmacological research and traditional chinese medicine (TCM) principles have gained widespread acceptance (Zhao et al., 2024; Poewe et al., 2017; Zheng et al., 2022). A review of TCM classics reveals that Gastrodia elata Bl. and Uncaria rhynchophylla (Miq.) Miq. ex Havil are frequently co-prescribed as a herbal pair (referred as Tianma-Gouteng) in formulations targeting symptoms similar to those of PD (Liu et al., 2015). For example, TCM preparations such as Gouteng Decoction, Tianma-Gouteng Granules, and Tianma Powder have demonstrated remarkable efficacy in treating various neurodegenerative disorders, including PD (Lei et al., 2025). These formulations not only alleviate the motor symptoms associated with PD but also improve cognitive function and quality of life for patients.

Historical records of TCM further corroborate the therapeutic efficacy of the Tianma-Gouteng. In “Lin Zheng Zhi Nan Yi An” (Case Records as a Guide to Clinical Practice), Ye Tianshi of the Qing dynasty documented a case of “liver wind ascending with splitting headache,” which was effectively treated using a combination of Tianma, Gouteng, *Haliotis diversicolor* Reeve (Shijue Ming), and *Paeonia lactiflora* Pall (Baishao), with symptom resolution observed after three doses. The esteemed Ming dynasty pharmacologist Li Shizhen described Tianma as “the herb that calms wind” and Gouteng as “the herb that quiets wind,” highlighting the remarkable efficacy of the Tianma-Gouteng in treating wind-related conditions. Contemporary pharmacological research has validated that Tianma components in Tianma, such as gastrodin and gastrodin glycoside, can modulate the PI3K/AKT pathway, reducing neuronal apoptosis and enhancing mitochondrial function, thus exerting neuroprotective effects and mitigating the progression of PD. Active components in Gouteng, including corynoxeine, isocorynoxeine, isorhynchophylline, and hirsuteine, have demonstrated the capacity to inhibit neuroinflammation and oxidative stress pathways, thereby alleviating the symptoms of PD. However, due to the limitations of current separation and identification technologies, as well as the diversity of active components and the intricate biological functions of TCM, they pose significant challenges to systematically and comprehensively elucidating the underlying mechanisms and potential therapeutic components of the Tianma-Gouteng in PD treatment using conventional analytical techniques.

This study employs network pharmacology, molecular docking, molecular dynamics simulations, and Density Functional Theory (DFT) calculations to thoroughly investigate the underlying mechanisms and potential components of Tianma-Gouteng in the regulation of PD.

The concept of “network pharmacology” was first introduced by British pharmacologist Andrew L. Hopkins in October 2007 (Hopkins, 2007), and since then, it has been recognized as a specialized branch of pharmacology. Network pharmacology plays a critical role in systems biology by bridging the gap between TCM theory and contemporary pharmacological research (Jiashuo et al., 2022). Through the application of network pharmacology, researchers can develop hierarchical analysis networks to systematically elucidate the multi-component and multi-target mechanisms of TCM. Utilizing this approach, this study systematically constructs the potential mechanisms by which the Tianma-Gouteng regulates PD (Zhang et al., 2023). Additionally, molecular docking and molecular dynamics simulation techniques are employed to facilitate a more comprehensive exploration of these mechanisms and to evaluate their stability (Gellner and McCann, 2016). This study also emphasizes the chemical characteristics of the plant’s active molecules, employing DFT calculations to investigate the potential reasons for their diverse biological activities from a quantum chemistry perspective.

The primary objectives of this study are delineated as follows:

1. Using network pharmacology methods, the active components and related targets of the Tianma-Gouteng are screened from databases such as TCMSP, HERB, SuperPred, and Swiss Target Prediction. An interaction network with PD-related targets is constructed, and its multi-target, multi-pathway therapeutic mechanisms and molecular binding patterns are explored through Protein-Protein Interaction (PPI) network analysis and the Metascape tool.
2. A preliminary investigation of the binding sites and binding affinity between the active components and target proteins is conducted using molecular docking techniques.
3. Molecular dynamics simulations are employed to further explore the binding mechanisms and stability of the interactions between the active components and target proteins.
4. Utilizing DFT, the quantum chemical structure of the active molecules is analyzed in greater detail, aiming to elucidate the potential mechanisms of their biological activity from the perspectives of energy characteristics and electronic structure.

In summary, this study integrates bioinformatics and computational chemistry strategies to comprehensively analyze the potential mechanisms and biological pathways of the Tianma-Gouteng in the treatment of PD, providing theoretical support and foundational insights for the development of novel therapeutic drugs for PD.

## 2 Materials and methods

### 2.1 Analysis of the network pharmacology study results of the Tianma-Gouteng medicinal pair in the treatment of PD

Utilizing network pharmacology methods, the study systematically examines the potential mechanisms of the Tianma-Gouteng in PD treatment, as detailed in Supplementary Table S1. Firstly, active ingredients of the Tianma-Gouteng were screened based on the TCMSP database, with oral bioavailability (OB ≥ 0.30) and drug-likeness (DL ≥ 0.18) serving as preliminary selection criteria. To improve prediction accuracy, further analysis of the active ingredients was conducted using the HERB database. Target prediction was carried out using the SuperPred, DrugBank, and STP platforms on the basis of Lipinski’s Rule of Five. To ensure data consistency, gene nomenclature was standardized using the UniProt database.

PD-related targets were obtained from the GeneCards, OMIM, PharmGKB, and DisGeNET databases using “Parkinson’s Disease” as the search term. Following data integration, redundant entries were eliminated. The targets associated with the medicinal pair were then intersected with the disease-related targets to identify common action targets. A “medicinal pair-component-target” regulatory network was constructed using Cytoscape 3.9.1 (Majeed and Mukhtar, 2023; Shannon et al., 2003). The Degree values for each component were calculated using the Network Analyzer plugin, facilitating the selection of key active components.

To further investigate the interactions among these common targets and identify core target sets, the common targets were imported into the STRING database (with a confidence score of ≥0.90, species: *Homo sapiens*) to construct a PPI network. Topological analysis was performed using the CytoNCA and CytoHubba plugins of Cytoscape, including seven metrics: degree centrality (DC), betweenness centrality (BC), cellular component (CC), maximal neighborhood component (MNC), subgraph centrality (SC), network centrality (NC), and eigenvector centrality (EC). The top 10 targets, as ranked by each metric, were selected for Venn analysis to identify the core target set. Additionally, a modular analysis of the PPI network was conducted using the MCODE plugin to identify key functional clusters, thereby revealing potential synergistic mechanisms.

This study used the Metascape platform to perform a multidimensional functional annotation of the common targets. In the Gene Ontology (GO) enrichment analysis, the parameters were specified as follows: the species was restricted to “*Homo sapiens*,” the significance threshold was set at *p* < 0.05, a minimum of three enriched genes was required, and multiple testing correction was applied using the Benjamini–Hochberg method. The analysis covered three dimensions: biological process (BP), with a primary focus on cascade reactions and regulatory networks involving the targets; molecular function (MF), which focused on protein binding properties and changes in enzyme activity; and cellular component (CC), which revealed the subcellular localization features of the common targets. For the Kyoto Encyclopedia of Genes and Genomes (KEGG) pathway enrichment analysis, in addition to the basic parameters mentioned above, an enrichment factor greater than 1.50 was set as a screening criterion. The analysis primarily focused on pathways associated with neurodegenerative diseases, dopaminergic synaptic regulation, and signaling pathways related to mitochondrial function. After the raw data were log10 (*p*-value) transformed, entries with smaller p-values were deemed significant and selected for further examination. Visualization was conducted using a bubble plot generated on the Microbio platform, where the size of the bubbles indicates the number of enriched genes, and the color gradient represents the significance level of the *p*-value.

### 2.2 Molecular docking

The PD-related receptor proteins identified in section 2.1.4 underwent preprocessing using Discovery Studio 2019, which included the repair of missing residues, optimization of protonation states, and removal of crystallization water molecules at non-binding sites. The semi-flexible docking method, using the CDOCKER module, was applied (Wu and Brooks, 2021), with residues within a 5–10 Å range of the co-crystal ligand defined as the active pocket to simulate the binding of the ligand to the receptor protein. Finally, the screening results were analyzed based on the system scoring function, and the best binding model was selected and visualized using PyMol (Seeliger and de Groot, 2010; Del Conte et al., 2023). The chosen binding mode was saved as a PDB complex structure file, serving as the initial conformation and input file for subsequent molecular dynamics simulations.

### 2.3 Molecular dynamics simulation

Based on the results from Sections 2.1, 2.2, molecular dynamics simulations were conducted on the selected best complex. Molecular dynamics simulations allow the analysis of molecular motion, interactions, and dynamic changes at the atomic level, providing insights into the stability, conformational changes, and dynamic behavior of protein-ligand binding. The researchers used the Gromacs 2025 Linux software to construct the simulation system, placing the receptor-ligand complex in a TIP3P water solvent model (Rosas Jimenez et al., 2024), ensuring that the box boundaries were at least 12 Å away from the complex to fully solvate the system and avoid boundary effects. The system charge was balanced using Na^+^ and Cl^−^, with the ionic concentration set to 0.154 M, corresponding to physiological saline concentration. To improve simulation accuracy, the AMBER99SB force field was used (Roterman et al., 2024), which is particularly suitable for describing non-bonded interactions and binding modes in protein-small molecule complexes.

The initial energy optimization was performed using a 5000-step steepest descent method, aiming to reduce unfavorable contacts and high-energy conformations in the system (Heid, 2023). The convergence threshold was set to 10 kJ (mol·nm)^−1^ to ensure effective adjustment of the force field parameters. Subsequently, a 2000-step conjugate gradient method was applied for fine optimization to further adjust the energy state of the system and ensure the structural integrity of the system (McCarthy, 1989).

During the equilibration phase, a 100 ps NVT ensemble simulation was first performed with a time step of 2 fs, gradually heating the system to 300 K to eliminate the influence of the initial structure and ensure thermodynamic equilibrium. Subsequently, an NPT ensemble simulation was conducted for 100 ps at a constant pressure of 1 bar, maintaining the system’s density and pressure stability to ensure the system reached a stable thermodynamic state in preparation for the subsequent production simulation. The formal molecular dynamics simulation was conducted for 100 ns, with the temperature maintained at 300 K and pressure kept at 1 bar. The time step for sampling was set to 2 fs, and the trajectory sampling interval was 10 ps. To ensure the stability and accuracy of the simulation, temperature, pressure, volume, and other physical properties were regularly monitored throughout the process to ensure they remained within the expected ranges.

### 2.4 DFT theoretical calculation

This study evaluates the chemical reactivity of the core components of the Tianma-Gouteng based on DFT. Firstly, the geometric structures were optimized at the ωB97XD/def2TZVP level of theory, using the Gaussian 09 Linux program. After optimization, single-point energy calculations were performed using the same basis set, and the wavefunction analysis was carried out using the Multiwfn_3.8 (Lu, 2024; Lu and Chen, 2012) program to analyze the energy distributions of the highest occupied molecular orbital (HOMO) and lowest unoccupied molecular orbital (LUMO), the electrostatic potential (ESP) surface distribution, Interaction Region Indicator (IRI), and Reduced Density Gradient (RDG) among other properties.

### 2.5 ADMET and BBB prediction

In this study, the absorption, distribution, metabolism, excretion, and toxicity (ADMET) properties of the compounds were predicted using the ADMET Descriptors module in BIOVIA Discovery Studio 2024. This module calculates a series of key pharmacokinetic parameters based on molecular structures, including aqueous solubility at 25 °C, blood-brain barrier permeability (BBB), cytochrome P450 2D6 inhibition (CYP2D6 inhibition), hepatotoxicity, human intestinal absorption (HIA), and plasma protein binding (PPB).

## 3 Results and discussion

### 3.1 Network pharmacology study results of the Tianma-Gouteng in the intervention of Parkinson’s disease

**FIGURE 1:**
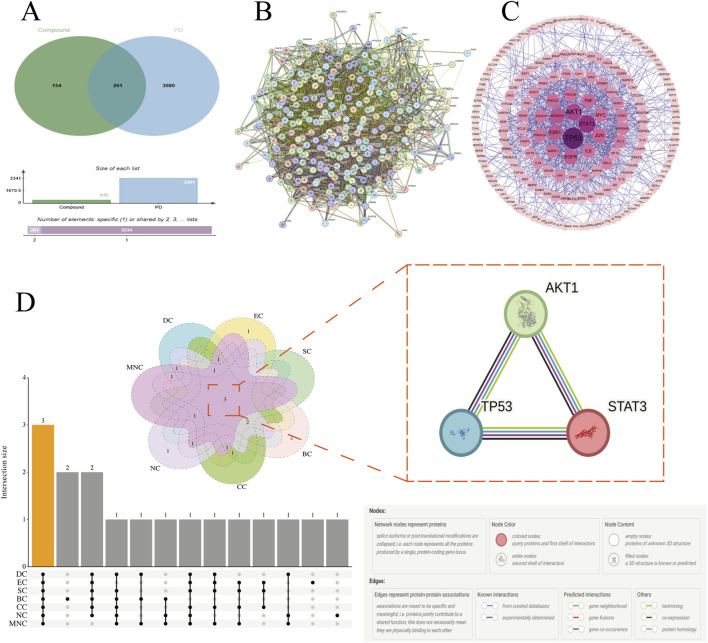
Network of common targets and identification of core targets; **(A)** Common targets for PD and “Tianma-Gouteng”; **(B)** 261 common targets PPI network from STRING database; **(C)** PPI network constructed using Cytoscape software for topological property analysis of network nodes; **(D)** Venn diagram analysis for screening core targets based on the intersection of seven topological properties.

**FIGURE 2:**
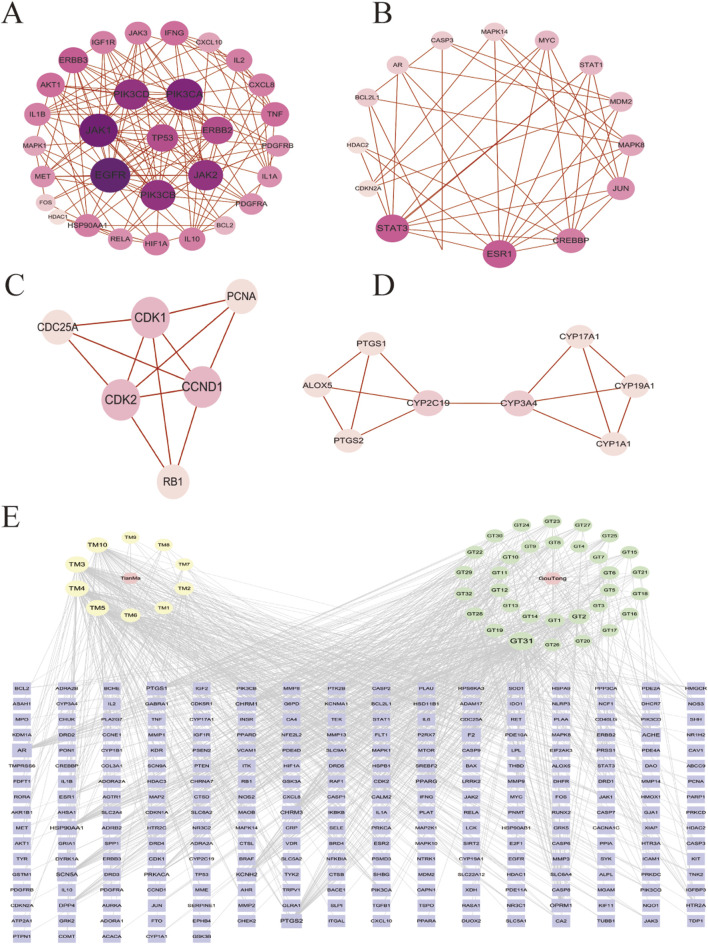
**(A)** Modular 1 consists of 30 nodes with 133 edges (cluster score = 9.17); **(B)** Modular 2 consists of 16 nodes with 41 edges (score = 5.46); **(C)** Modular 3 consists of 6 nodes with 12 edges (score = 4.80); **(D)** Modular 4 consists of 8 nodes with 13 edges (score = 3.71); **(E)** “medicinal pair-component-target” regulatory network. The size and color of the nodes were positively correlated with the target’s degree of association.

Network pharmacology analysis revealed that Tianma-Gouteng intervenes in the progression of PD through a synergistic multi-target mechanism. A total of 42 active compounds and 416 corresponding targets were identified; by intersecting with known PD-related targets, 261 common targets were obtained (Figure 1A). Notably, 60% of these targets are associated with known PD-related pathological genes, indicating the herb pair’s multi-target regulatory potential. Regulatory network analysis (Figure 2E) revealed that compounds such as quercetin (QU) and kaempferol (KA) exhibit notable network centrality (Table 1). Their established neuroprotective activities are consistent with the results predicted by the network analysis (Gunesch et al., 2020). Notably, QU, the compound with the highest degree centrality (Degree = 109), has been extensively studied and shown to exert potential therapeutic effects by modulating multiple key mechanisms involved in PD pathogenesis, including neuroprotection, inflammatory response, and inhibition of PD-related proteins (Chen et al., 2022; Zhu et al., 2013).

**TABLE 1:** Key potential components of “Tianma-Gouteng” for regulating PD.

Abbreviation	Compound name	Compound name	Degree
QU	Quercetin	MOL000098	109
HY	4-(4′-hydroxybenzyloxy)benzyl methyl ether	5318157	76
KA	Kaempferol	MOL000422	76
HE	20-Hexadecanoy lingenol	MOL011455	48

PPI network analysis further identified p-AKT1 (PDB ID: 1UNQ; UN), P53 (PDB ID: 2MWP; MW), and STAT3 (PDB ID: 5U5S; US) as core targets (Figures 1B–D; Table 2). These proteins play crucial roles in apoptosis, oxidative stress, and neuroinflammation associated with PD (Wang et al., 2024; Yu et al., 2024; Kou et al., 2022). Modular analysis further validated the biological significance of the core targets, with the PPI network divided into nine functional modules (Supplementary Table S2). Four top-scoring modules were visualized (Figures 2C–D), with module 1 (Figure 2A) containing the core targets AKT1 and P53 (Z et al., 2021), and module 2 (Figure 2B) containing STAT3 (Xu et al., 2021). AKT1 is located at the core of the PI3K-AKT signaling pathway (Ersahin et al., 2015), P53 is closely associated with mitochondrial dysfunction, and STAT3 is a key regulator of neuroinflammation. Together (Lashgari et al., 2021), these three targets form a multidimensional regulatory network for intervening in PD.

**TABLE 2:** Analysis of the network topological parameters of key targets.

Abbreviation	Gene name	PDB ID	Network topological parameter						
			Betweenness	Closeness	MNC score	Network	Degree	Eigenvector	Subgragh
UN	AKT1	1UNQ	3,420.61	0.09	37	19.68	38	0.22	104
MW	TP53	2MWP	7,803.37	0.09	55	38.65	57	0.29	63
US	STAT3	5U5S	4,284.26	0.09	39	22.52	43	0.25	61

**FIGURE 3:**
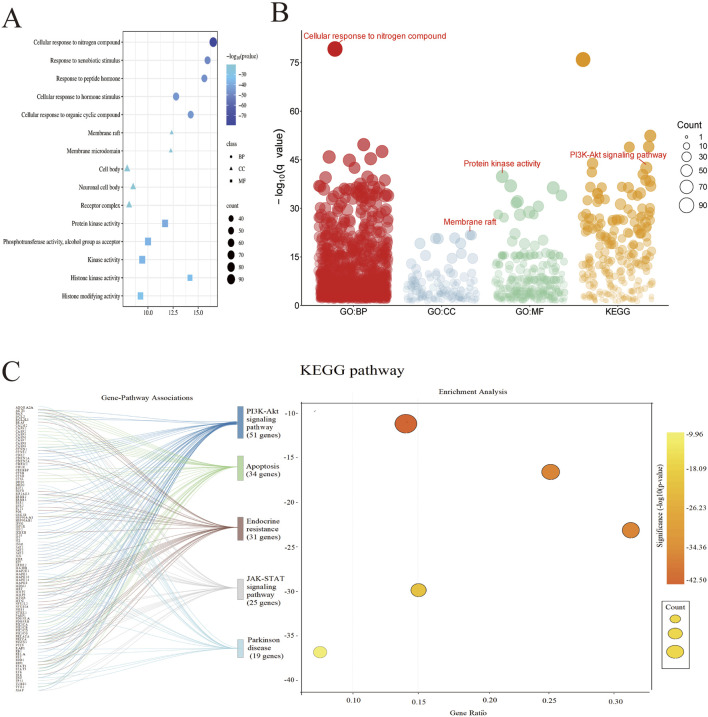
**(A)** GO enrichment analysis circles represent biological processes (BP), triangles denote cellular components (CC), and squares indicate molecular functions (MF); **(B)** Gene set enrichment analysis; **(C)** KEGG pathway enrichment analysis.

Gene set enrichment analysis revealed the potential mechanisms of action of the herb pair from a systems level (Figure 3B). GO enrichment analysis (Figure 3A) showed that the targets were significantly enriched in functional categories closely related to PD pathology, such as “response to nitrogen compounds” (GO:1901699) and “membrane rafts” (GO:0045121). The regulation of “protein kinase activity” (GO:0004672) may explain its role in improving protein abnormal phosphorylation. In the KEGG pathway enrichment analysis (Figure 3C), the PI3K-AKT signaling pathway (HSA04151) showed strong enrichment significance. AKT1 in this pathway can inhibit the kinase activity of GSK3β by phosphorylating it, thereby reducing α-synuclein aggregation and enhancing mitochondrial function (Goyal et al., 2023).

Integrating network topological features and functional enrichment results, this study proposes that the Tianma-Gouteng may exert therapeutic effects through the following synergistic mechanisms: Active compounds activate the AKT1-mediated PI3K-AKT pathway (Jin et al., 2022), inhibiting the overactivation of GSK3β; regulate STAT3 expression to improve the neuroinflammatory microenvironment; balance P53 to promote the expression of anti-apoptotic factors and protect dopaminergic neurons. Additionally, it targets membrane raft structures to regulate neurotransmitter receptor function (Long et al., 2021).

### 3.2 Molecular docking Verification

**FIGURE 4:**
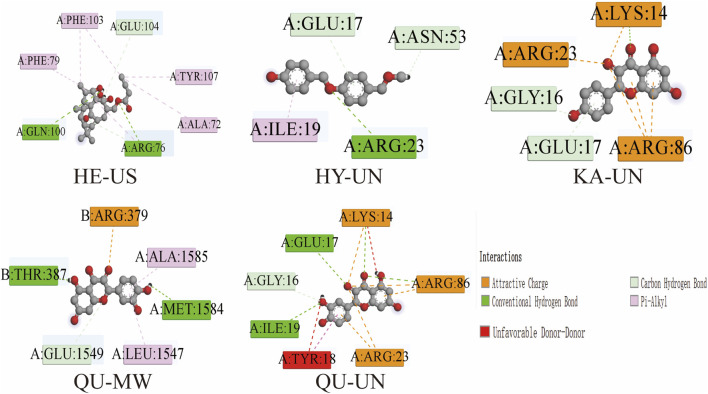
Five molecular interaction diagrams (HE-US, HY-UN, KA-UN, QU-MW, and QU-UN) depict a central molecule surrounded by labeled residues. Color-coded dashed lines denote interaction types—attractive charge (orange), carbon hydrogen bond (green), conventional hydrogen bond (blue-green), pi-alkyl (pink), and unfavorable donor-donor (red)—as indicated in the key. Each diagram features a distinct combination of interacting residues and bond types.

Molecular docking analysis was used to study the binding affinity and binding modes between the core components and the three core target proteins, providing in-depth structural information. As shown in Figure 4, the four core components exhibited significant binding with their corresponding target proteins. QU-UN and QU-MW show the binding modes between the compound QU and the target proteins UN (AKT1) and MW (TP53), respectively. In the interaction with the AKT1 target, the binding of QU to AKT1 involves multiple hydrogen bonds, particularly with residues GLU17, GLY16, ILE19, THR387, GLU1594, and MET1584, which form significant hydrogen bond interactions with the QU molecule. Additionally, the residues LYS14, ARG86 (23), ARG379, ALA1585, and LEU1574 contribute to the binding with QU through dispersion forces, further enhancing the binding affinity between them.

KA-UN and HY-UN illustrate the binding modes of compounds KA and HY with the AKT1 target protein. The results indicate that the binding of KA to AKT1 primarily relies on dispersion interactions, with particularly strong interactions observed at the ARG86 (23), ILE19 residue. Hydrogen bond interaction is provided by the GLY16, GLU17, ASN53, and ARG23 residues.

HE-US reveals that compound HE binds STAT3 via key residues (ARG76, GLN100, PHE103) through hydrogen bonds and van der Waals forces. Given STAT3’s role in driving neuroinflammation—where it promotes pro-inflammatory cytokine transcription (IL-6, TNF-α, IL-1β) and activates microglia and astrocytes—this binding suggests HE may inhibit STAT3 phosphorylation and nuclear translocation. This would suppress cytokine release, reduce glial activation, break the neuroinflammatory cycle, and protect dopaminergic neurons. The finding links STAT3 to neuroinflammation and provides a molecular basis for the Tianma-Gouteng herb pair’s anti-PD effects.

### 3.3 Analysis of molecular dynamics simulation results

To gain deeper insights into the molecular mechanisms of the binding between core components and core target proteins, the researchers performed 100 ns molecular dynamics simulations on the complexes QU-UN, QU-MW, KA-UN, HE-US, and HY-UN, and evaluated the stability of the system’s structure during the simulation using root mean square deviation (RMSD) (Cohen and Sternberg, 1980), root mean square fluctuation (RMSF) (Peterson et al., 2017), radius of gyration (Rg) (Ahmed et al., 2020), and hydrogen bonds (Hbond) (Bitencourt-Ferreira et al., 2019).

**FIGURE 5:**
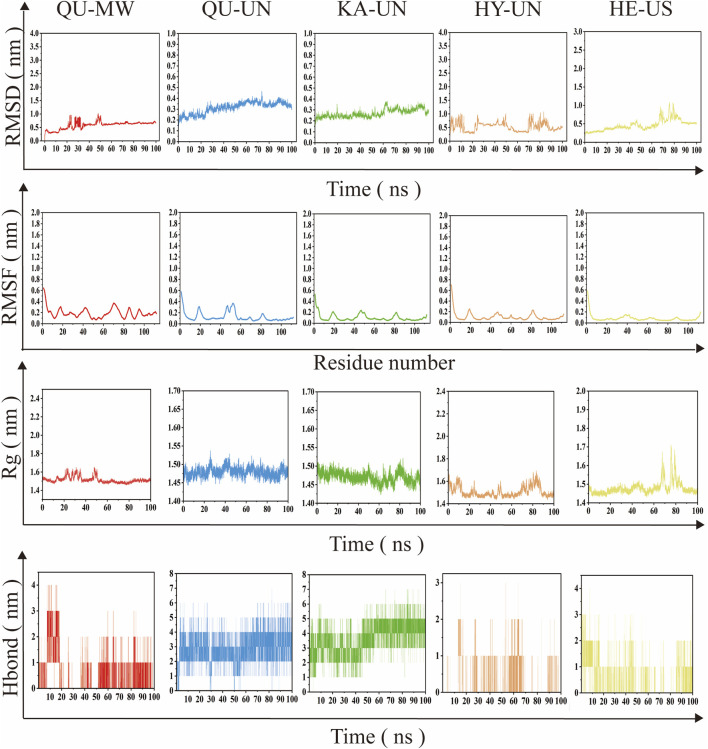
Molecular dynamics simulation of the complex system; Change of RMSD of the complex system over the simulation time. RMSF of the amino acid residues of the complex system over a 100 ns simulation time. Change in the radius of gyration of the complex system over the simulation time. Change in the number of hydrogen bonds in the complex system over the simulation time.

The RMSD of the protein backbone atoms during the simulation is shown in Figure 5. During the 100 ns molecular dynamics simulation, the five complexes showed significant differences; the RMSD values of the QU-UN, QU-MW, and KA-UN complexes remained stable at lower levels, whereas the HY-UN and HE-US complexes exhibited significant fluctuations, with the RMSD of HY-UN continuously oscillating throughout the simulation and never reaching equilibrium, suggesting that these two complexes exhibit significant conformational instability.

The local flexibility of the target protein residues was further investigated through RMSF analysis. The RMSF peaks of the α-carbon atoms for all complexes were below 0.4 nm, reflecting the overall rigidity of the protein backbone. The QU-UN complex exhibited specific fluctuation peaks at residues 20 and 45 (RMSF >0.35 nm); the KA-UN complex showed three significant peaks near residues 20, 50, and 80. Molecular docking results indicated that these highly fluctuating residues are located within the ligand-binding pocket, suggesting that ligand binding may induce local conformational perturbations through steric hindrance or electrostatic interactions, thereby affecting the mobility of key residues.

The Rg analysis revealed changes in the compactness of the complex’s tertiary structure. The Rg values of the QU-UN and QU-MW complexes remained stable (fluctuation range <0.02 nm), confirming that the embedding of ligand QU did not disrupt the overall protein fold. The KA-UN complex exhibited slight fluctuations (ΔRg ≈0.05 nm); this phenomenon corresponds to the slight drift in RMSD and the local peaks in RMSF, collectively indicating moderate conformational adjustments induced by ligand binding; whereas the Rg values of the HY-UN and HE-US complexes exhibited large oscillations (ΔRg >0.1 nm), further corroborating the overall instability observed in the RMSD analysis.

The number of hydrogen bonds between the protein and ligand directly reflects the stability of the binding interface. The QU-UN complex formed 2-4 stable hydrogen bonds. The KA-UN complex-maintained 5 high-number hydrogen bonds after 50 ns, suggesting strong binding affinity. The QU-MW complex ultimately stabilized to form a single hydrogen bond. In contrast, the HY-UN and HE-US complexes exhibited drastic fluctuations in the number of hydrogen bonds (instantaneous minimum value of 0).

Multivariate analysis confirmed that the QU-UN, QU-MW, and KA-UN complexes exhibited excellent conformational stability and interaction strength, while the HY-UN and HE-US complexes were excluded from further study due to abnormal consistency across multiple indicators.

### 3.4 Principal component analysis and MM-PBSA analysis of the composite system

MM-PBSA (Molecular Mechanics Poisson-Boltzmann Surface Area) calculations were performed to determine the binding energies of the QU-UN, QU-MW, and KA-UN complexes to further understand the receptor-ligand interaction mechanism (Supplementary Figure S1). presents the total energy of the three complexes, all of which exhibit low binding free energies, indicating strong binding affinity between the ligand and receptor protein, which is consistent with the molecular docking results. The free energy decomposition table for the residues (Supplementary Tables S3–S5) reveals that in the QU-UN complex, Electrostatic interactions contribute a strong negative value (−119.8 kcal mol^-1^), which is the primary driving force. This is primarily due to the interaction between QU and residue ARG86 (ΔG = −8.158 kcal mol^-1^). In the QU-MW complex, hydrophobic interactions are the core driving force stabilizing the receptor-ligand binding. In the KA-UN complex, the strong hydrogen bonding interaction between ligand KA and residue ARG23, LEU52 stabilizes their binding.

Principal Component Analysis (PCA) is a multivariate data analysis technique that effectively reveals the details of biomolecular motion, and the Free Energy Landscape (FEL) constructed based on PCA can explain the system’s stable states (energy wells) and transition states (energy barriers), helping to understand the energy barriers and dynamic pathways associated with conformational changes. PC1 and PC2 represent the first principal component (PC1, RMSD) and the second principal component (PC2, Rg) of the complex, respectively. These two principal components are the main feature directions extracted by the program from high-dimensional data, which can visually describe and visualize the complex structural and dynamic information of the complex in molecular dynamics simulations. The regions with blue and purple tones in the figure indicate that the stable conformation of the complex formed by the active ingredient and target protein can be depicted at a lower energy within the minimum free energy region. If the interaction between the protein and ligand is weak or unstable, the free energy landscape will show multiple, rough-surfaced minima and energy clusters. Conversely, strong and stable interactions can form a nearly single, smooth energy cluster in the potential energy distribution. The free energy landscape of the three protein-ligand complexes formed a nearly single, sharp minimum energy cluster, indicating strong and stable interactions between the active ingredient and the target protein, ensuring the stability of the complex.

### 3.5 DFT theoretical calculation results

**FIGURE 6:**
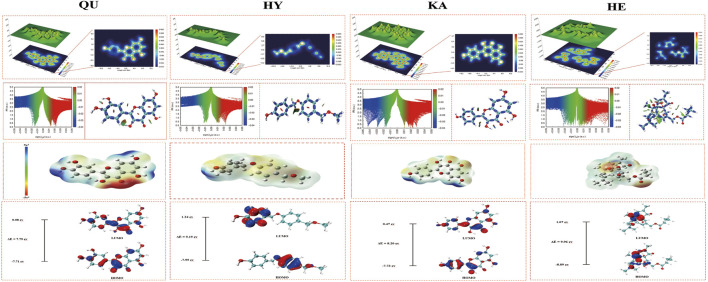
DFT theoretical calculations of the compound; The first row presents the 3D and 2D distributions of LOL for the four core components.; The second row presents the IRI scatter plots and RDG spatial maps of the four core components, with blue regions representing hydrogen bonds, green regions indicating van der Waals interactions, and red regions denoting steric repulsion; The third row presents the electron density maps of the four core components, with red regions indicating electron-rich areas and blue regions representing electron-deficient areas; The fourth row illustrates the HOMO and LUMO orbital distributions and energy gaps of the four core components, with red and blue representing the positive and negative wave functions, respectively, without physical significance.

This study systematically analyzes the chemical properties of four core natural products from a quantum mechanical perspective using DFT calculations (Figure 6). The study focuses on electron density (Landeros-Rivera et al., 2022), electrostatic potential distribution (Wada and Nakamura, 1981), weak interaction analysis (RDG/IRI), and frontier molecular orbital characteristics (Alblozy et al., 2025).

The electron density and electrostatic potential (ESP) distribution further reveal the reactive sites and interaction types of the molecule during its interaction with proteins. The hydroxyl and carbonyl regions of QU and KA molecules exhibit strong negative electrostatic potential, showing distinct electron-rich characteristics, making them potential hydrogen bond acceptors; while the nearby electrophilic hydrogen atoms (positive ESP regions) act as hydrogen bond donors. This charge complementarity forms a stable multi-point hydrogen bond network, facilitating precise docking in the protein binding pocket.HY and HE, on the other hand, use ether bonds, hydroxyl groups, and carbon-carbon double bonds as their main reactive centers. Combined with their longer alkyl chains and hydrophobic backbones, they participate in hydrogen bond formation during binding, while also enhancing affinity through electrostatic attraction and van der Waals forces. For example, the flexible alkyl chain of HY can insert into the hydrophobic pocket region, forming a stable hydrophobic enclosure.

RDG/IRI visualization reveals weak intermolecular interactions within the molecule. To further analyze non-covalent interactions and conformational stability, this study introduces the RDG and IRI methods. RDG reveals the types and spatial locations of weak interactions, such as van der Waals forces, hydrogen bonds, and π-π interactions, through the distribution of electron density and its gradient. IRI, on the other hand, captures weak interaction regions in space with greater sensitivity, making it particularly suitable for intermolecular interface analysis.

The analysis shows that QU and KA molecules contain strong intramolecular hydrogen bonds (O-H···O=C, RDG ≈0.03 a.u.) and π-π conjugation between aromatic rings; these interactions promote the molecule to adopt a specific folded conformation, achieving preorganization, reducing the conformational rearrangement free energy required for binding, thereby enhancing the entropic benefit of binding to the target protein. In contrast, HY and HE are primarily dominated by dispersion forces and van der Waals interactions (RDG ≈0.5 a.u.), and their flexible structure provides higher conformational adjustability during binding, facilitating adaptation to diverse protein binding pockets, but potentially accompanied by conformational entropy loss.

HOMO-LUMO energy gap and chemical reactivity. Frontier Molecular Orbitals analysis shows that all four compounds have a small HOMO-LUMO energy gap. The HOMO-LUMO energy gap reflects the minimum energy required for a molecule to transition from the ground state to the excited state, serving as an important quantitative indicator of its electronic reactivity and stability. The smaller the energy gap, the more easily the molecule undergoes charge transfer, exhibiting higher chemical reactivity.

The HOMO orbital density of QU and HY is primarily distributed over the hydroxyl-substituted benzene ring and pyran ring, representing their nucleophilic active centers, while the LUMO is distributed over electron-accepting regions such as the benzopyran ring, serving as a potential electrophilic site. The extremely low energy gap facilitates efficient electron transfer from the HOMO region to the LUMO region, inducing rearrangement of the electron cloud at the active sites of the target protein (such as Ser, Thr, and other amino acids with polar side chains), enhancing the hydrogen bond network and stabilizing the binding conformation. The HOMO → LUMO transition of HE is characterized by the electron transfer from the electron-donating benzene ring through the ether bond to the electron-accepting benzene ring, its prominent π-delocalized structure favors π-π stacking or cation-π interactions with aromatic residues (such as PHE and TYR) in the protein, thereby synergistically strengthening the non-covalent binding.

### 3.6 Pharmacokinetic prediction

The pharmacokinetic prediction results of the four compounds (QU, KA, HY, HE) (Table 3) reveal differences in metabolic stability and central nervous system (CNS) penetration. In terms of blood-brain barrier (BBB) permeability, all compounds exhibit BBB scores ranging from 2 to 3, indicating low to moderate CNS penetration. Specifically, QU, KA, and HE have a BBB score of 3 (low permeability), suggesting limited ability to cross the BBB and potentially exerting anti-PD effects primarily via peripheral targets such as AKT1 and TP53. Additionally, all compounds show negative plasma protein binding (PPB) values, implying a relatively high free drug concentration in plasma, which may enhance *in vivo* pharmacological activity.

**TABLE 3:** Pharmacokinetic prediction.

Drug	Molecular formula	Molecular weight	Hepatotoxicity	PPB	BBB
QU	C₁₅H₁₀O₇	302.245	1.65573	−7.33887	3
KA	C₁₅H₁₀O₆	286.245	1.40834	−4.6066	3
HY	C₁₅H₁₆O₃	244.293	−5.67466	−2.84143	2
HE	C₂₄H₃₄O₆	418.536	−11.3049	−6.83298	3

## 4 Conclusion

This integrative computational study elucidates the multi-target mechanism of the Tianma-Gouteng herb pair in combating Parkinson’s disease (PD). Through network pharmacology, we identified potential targets AKT1, STAT3, and TP53, which may regulate the PI3K-AKT pathway, neuroinflammation, and apoptosis—key processes involved in the onset and progression of PD. Molecular docking revealed that quercetin (QU) and kaempferol (KA) exhibit high-affinity binding to AKT1 and TP53 via hydrogen bonds, while the hyodeoxycholic acid (HY) and 20-hexadecanoylingenol (HE) complexes demonstrated relatively weaker binding. Molecular dynamics simulations further confirmed that the complexes formed by QU, KA, and HY with AKT1 and TP53 exhibit excellent stability and sustained interactions over 100 ns trajectories, with RMSD values consistently below 0.2 nm and stable hydrogen bonding patterns.

Density functional theory calculations using the ωB97X-D/def2-TZVP level of theory revealed that QU and KA possess superior electronic properties, including lower HOMO-LUMO gaps (QU: 7.79 eV; KA: 8.20 eV) and well-defined hydrogen bond donor/acceptor regions in their electrostatic potential maps, enabling effective binding to the targets.

Based on these computational findings, we predict that the three core compounds may exert neuroprotective effects by modulating the phosphorylation states of AKT1 and TP53, though this requires experimental validation.

Pharmacokinetic predictions using Discovery Studio 2024 revealed important translational considerations: QU (BBB level 3), KA (BBB level 3), and HY (BBB level 2) exhibit low to moderate blood-brain barrier permeability, indicating limited passive diffusion into the central nervous system. This finding tempers the direct interpretation of these compounds as CNS-penetrant drug candidates. Thus, QU, KA, and HY might contribute to PD therapeutic effects through peripheral pathways, particularly in early intervention or adjunctive treatment strategies.

In summary, this study demonstrates that Tianma-Gouteng exerts potential anti-PD effects through the synergistic modulation of AKT1 and TP53. As key stable components of this herb pair, QU, KA, and HY exhibit favorable binding properties and electronic characteristics. However, the blood-brain barrier permeability limitations identified for these compounds highlight the need for further optimization, such as structural modification to enhance CNS penetration, development of novel delivery systems, or exploration of their peripheral mechanisms of action. Future studies should combine experimental validation with ADMET optimization to fully realize the therapeutic potential of these natural compounds for PD treatment.

## Data Availability

The original contributions presented in the study are included in the article/Supplementary Material, further inquiries can be directed to the corresponding author.

## Glossary

PD: Parkinson’s disease
MD: molecular dynamics
DFT: Density functional theory
TCM: traditional chinese medicine
DC: degree centrality
BC: betweenness centrality
CC: cellular component
MNC: maximal neighborhood component
SC: subgraph centrality
NC: network centrality
EC: eigenvector centrality
KEGG: Kyoto Encyclopedia of Genes and Genomes
GO: Gene Ontology
HOMO: highest occupied molecular orbital
LUMO: lowest unoccupied molecular orbital
ESP: electrostatic potential
IRI: Interaction Region Indicator
RDG: Reduced Density Gradient
QU: quercetin
KA: kaempferol
HY: 4-4′-hydroxybenzyloxy
HE: 20-hexadecanoylingenol
MW: 2MWP
US: 5U5S
UN: 1UNQ
QU-UN: quercetin-1UNQ
QU-MW: quercetin-2MWP
KA-UN: kaempferol-1UNQ
HY-UN: 4-4′-hydroxybenzyloxy-1UNQ
HE-US: 20-hexadecanoylingenol-5U5S
RMSD: root mean square deviation
RMSF: root mean square fluctuation
Rg: radius of gyration
Hbond: hydrogen bonds
MM-PBSA: Molecular Mechanics Poisson-Boltzmann Surface Area
