# Supplementary material for: Investigating the molecular mechanisms of the “Tianma-Gouteng” herb pair in treating Parkinson’s disease: a bioinformatics approach and density functional theory with molecular dynamics simulations validation
Source: Front Bioinform. 2026 Apr 1;6:1796216. doi: 10.3389/fbinf.2026.1796216 (PMC13079657; doi:10.3389/fbinf.2026.1796216)
Supplement: Supplementary file 1 [file Table1.docx]

**Investigating the molecular mechanisms of the "Tianma-Gouteng" herb pair in treating Parkinson's Disease: a bioinformatics approach and density functional theory with molecular dynamics simulations validation**

Liping Zhou ^a,1^, Chenyang Fei ^b,1^, Quanxia Liu ^a, *^

^a^ Ningxia Medical University General Hospital, 804 Shenli Street, Yinchuan, 750004, China

^b^ Ruihe Digital Technology Co., Ltd. (Shenzhen), 19th Floor, West Wing, Skyworth Semiconductor Design Building, 18 Gaoxin South 4th Road, Nanshan District, Shenzhen, 518000, Chin

^1^ The authors made the same contribution to this work and share the role of the first author.

^*^Correspondence authors: E-mail addresses: 19995104086@163.com (Quanxia Liu)

**Table of Contents**

| 1. | Supplementary Table. 1 General Table of Databases and Their URLs | S3 |
| --- | --- | --- |
| 2. | Supplementary Table. 2 Cluster information of the common targets for TCM and diseases | S3 |
| 3. | More details of the MM/PBSA simulation | S4 |

**1. General Table of Databases and Their URLs**

| Supplementary Table. 1 General Table of Databases and Their URLs | | |
| --- | --- | --- |
| Abbreviation Name | Full name | Website address |
| Cytoscape | Cytoscape: a software environment for integrated models of biomolecular interaction networks | https://cytoscape.org/ |
| UniProt | **The Universal Protein Knowledgebase** | https://www.uniprot.org |
| GeneCards | The Human Gene Database | https://www.genecards.org/ |
| DAVID | Database for Annotation, Visualization, and Integrated Discovery | https://davidbioinformatics.nih.gov/ |
| PharmGKB | Pharmacogenetics and Pharmacogenomics Knowledge Base | https://www.pharmgkb.org/ |
| STRING | STRING Protein-Protein Interaction Networks Functional Enrichment Analysis | https://string-db.org/ |
| Bioinformatics | [**A free online platform for data visualization and graphing**](https://journals.plos.org/plosone/article?id=10.1371/journal.pone.0294236) | https://www.bioinformatics.com.cn |
| DISGENET | A comprehensive knowledge database integrating and standardizing information on disease associated genes and variants. | https://www.disgenet.com |
| TCMSP | Traditional Chinese Medicine Systems Pharmacology Database and Analysis Platform | http://lspnwu.edu.cn/tcmspphp |
| HERB | A high-throughput experiment- and reference-guided database of traditional Chinese medicine | http://herb.ac.cn |

**2. Cluster information of the common targets for TCM and diseases**

| Supplementary Table. 2 Cluster information of the common targets for TCM and diseases | | | | |
| --- | --- | --- | --- | --- |
| Cluster | Score | Nodes | Edges | Node IDs |
| 1 | 9.17 | 30 | 133 | RELA, MET, MAPK1, PIK3CD, AKT1, ERBB3, IGF1R, IFNG, JAK2, PIK3CB, CXCL8, IL2, TNF, PDGFRB, HDAC1, PDGFRA, IL10, TP53, HIF1A, EGFR, HSP90AA1, FOS, JAK1, PIK3CA, IL1B, JAK3, CXCL10, ERBB2, IL1A, BCL2 |
| 2 | 5.46 | 16 | 41 | MAPK8, STAT3, TYK2, CASP3, ESR1, BCL2L1, MDM2, STAT1, HDAC2, CREBBP, MAPK14, CDKN2A, JUN, MYC, CASP9, AR |
| 3 | 4.80 | 6 | 12 | RB1, PCNA, CDK1, CDK2, CCND1, CDC25A |
| 4 | 3.71 | 8 | 13 | CYP17A1, ALOX5, CYP2C19, CYP19A1, CYP1A1, PTGS2, CYP3A4, PTGS1 |
| 5 | 3.20 | 6 | 8 | NFKBIA, CAV1, IKBKB, CHUK, HSP90AB1, NOS3 |
| 6 | 3 | 3 | 3 | CASP7, CASP8, XIAP |
| 7 | 3 | 3 | 3 | CCNE1, CDKN1A, E2F1 |
| 8 | 3 | 3 | 3 | RAF1, BRAF, PRKACA |
| 9 | 3 | 3 | 3 | DHCR7, FDFT1, HMGCR |

**3. More details of the MM/PBSA simulation**

The following is the energy decomposition analysis for three sets of molecular dynamics (MD) simulations. We used the Generalized Born model (GB) to calculate the solvation energy and applied the idecomp=2 setting (indicating residue-level decomposition, including 1-4 interactions for electrostatic and van der Waals terms). The data include the complex, receptor, ligand, and the total energy decomposition of the binding free energy difference. Each part includes several energy terms: molecular mechanical energy (△GGAS), van der Waals energy (△VDWAALS), electrostatic energy (△EEL), polar solvation energy (△EGB), non-polar solvation energy (△ESURF), solvation energy (△GSOLV), and total energy (△TOTAL).


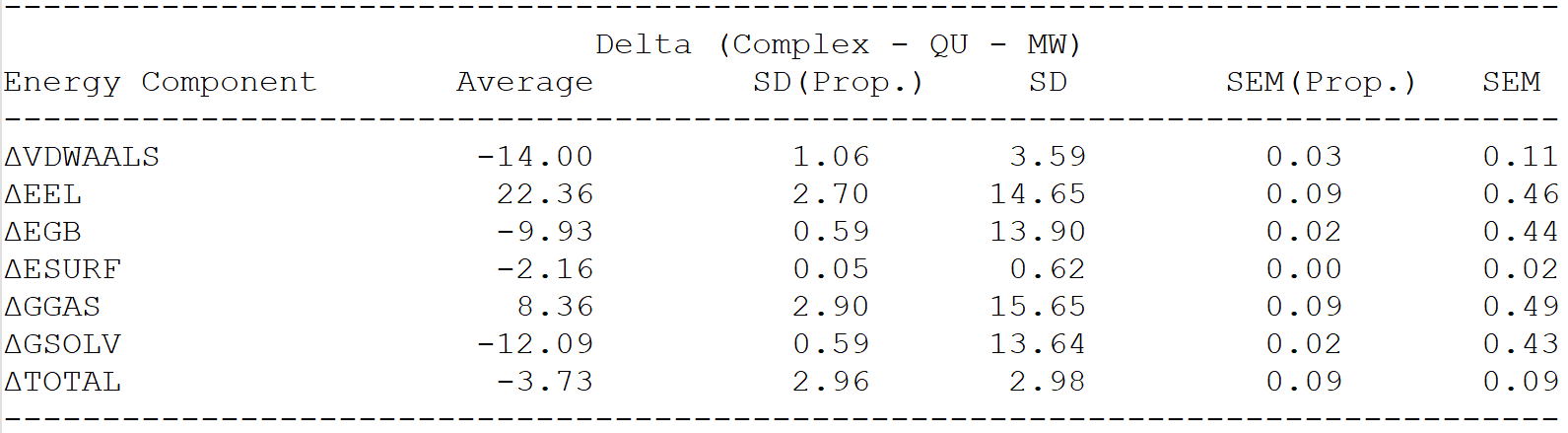


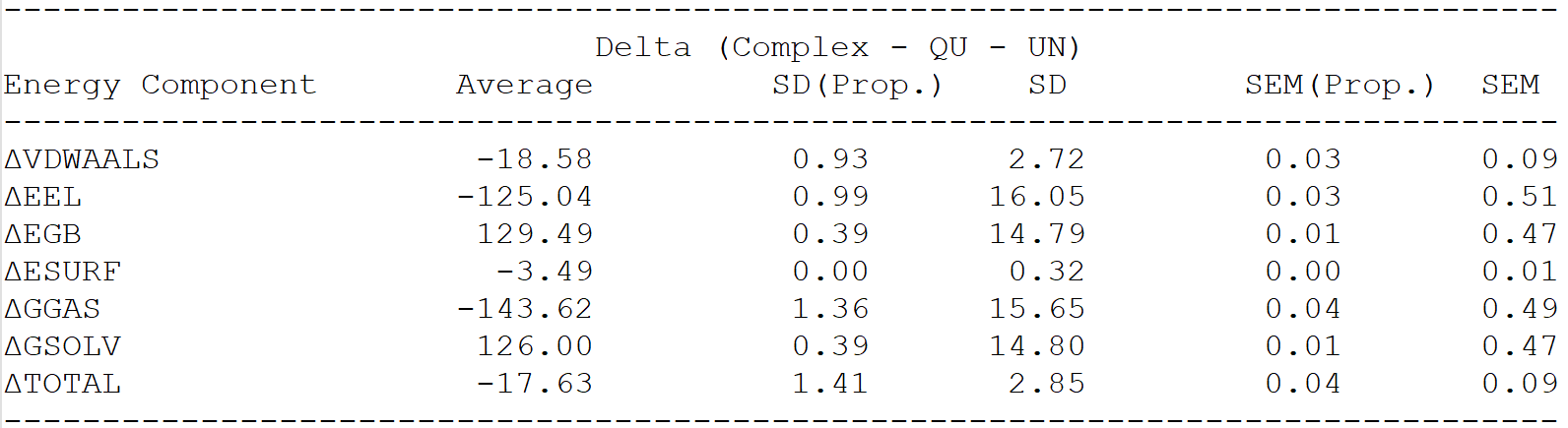


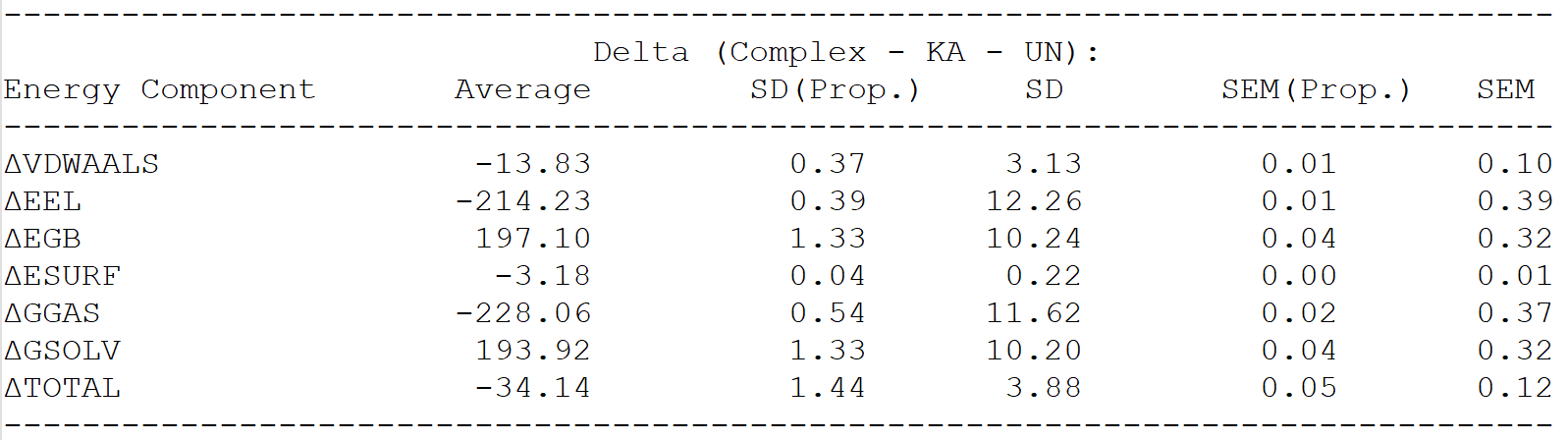


The table below summarizes the binding free energy contributions (ΔG) of core residues in each system, including the average (Avg.), standard deviation (Std. Dev.), and standard error of the mean (Std. Err.) .Statistical significance is based on

| Supplementary Table. 3 KA-UN System | | | | |
| --- | --- | --- | --- | --- |
| **Residue** | **ΔG (kcal•mol^-1^)** | **Std.Dev.** | **Std.Err.** | Whether it is significant |
| QU | **-9.342** | **11.145** | **0.352** | YES |
| **R:A:ARG:23** | **-10.320** | **12.274** | **0.388** | YES |
| **R:A:LEU:52** | **-2.028** | **5.658** | **0.179** | YES |
| **R:A:LYS:14** | **+0.226** | **17.665** | **0.558** | **NO** |
| **R:A:GLY:16** | **+0.018** | **3.864** | **0.122** | **NO** |
| **R:A:GLU:17** | **+0.107** | **23.588** | **0.746** | **NO** |
| **R:A:TYR:18** | **+0.012** | **6.222** | **0.197** | **NO** |
| **R:A:ILE:19** | **+0.002** | **5.408** | **0.171** | **NO** |
| **R:A:ASN:53** | **-0.644** | **8.677** | **0.274** | **NO** |
| **R:A:PHE:55** | **-0.022** | **5.332** | **0.169** | **NO** |

| Supplementary Table. 4 QU-UN System | | | | |
| --- | --- | --- | --- | --- |
| **Residue** | **ΔG (kcal•mol^-1^)** | **Std.Dev.** | **Std.Err.** | Whether it is significant |
| **QU** | **-4.880** | **13.241** | **0.419** | YES |
| **R:A:ARG:86** | **-8.158** | **14.211** | **0.449** | YES |
| **R:A:ASN:54** | **-1.262** | **9.083** | **0.287** | YES |
| **R:A:PHE:55** | **-1.703** | **5.241** | **0.166** | YES |
| **R:A:LYS:14** | **+0.127** | **27.041** | **0.855** | **NO** |
| **R:A:GLN:79** | **-0.372** | **7.477** | **0.236** | **NO** |

| Supplementary Table. 5 QU-MW System | | | | |
| --- | --- | --- | --- | --- |
| **Residue** | **ΔG (kcal/mol)** | **Std.Dev.** | **Std.Err.** | Whether it is significant |
| **QU** | **-12.124** | **10.881** | **0.344** | YES |
| **R:A:LEU:1547** | **-0.415** | **5.109** | **0.161** | YES |
| **R:A:SER:1548** | **-0.256** | **8.880** | **0.281** | YES |
| **R:A:GLU:1549** | **+0.04** | **27.313** | **0.863** | **NO** |
| **R:A:GLU:1551** | **+0.002** | **20.614** | **0.652** | **NO** |
| **R:B:ARG:379** | **-0.241** | **18.045** | **0.570** | YES |
| **R:B:PHE:385** | **-0.751** | **6.850** | **0.217** | YES |
| **R:B:THR:387** | **+0.030** | **19.479** | **0.616** | **NO** |
